# 711. High Prevalence of Positive Cell Cytotoxicity Neutralization Assay in Symptomatic and Asymptomatic Pediatric Oncologic Patients

**DOI:** 10.1093/ofid/ofad500.773

**Published:** 2023-11-27

**Authors:** David Zhang, Ronda Oram, Meghan Landry, Madan Kumar, Katie Cherny, Addison Hillerbrand, Larry K Kociolek, Nathalia Davila, Mariel Galvan, Karyssa Knopoff, Rasheed Ansari

**Affiliations:** University Hospitals, Cleveland, Ohio; Advocate Lutheran General, Park Ridge, Illinois; University of Chicago, Chicago, Illinois; University of Chicago Medicine, Chicago, Illinois; Northwestern University, Chicago, Illinois; Northwestern Medicine, Chicago, Illinois; Ann & Robert H. Lurie Children's Hospital of Chicago, Chicago, IL; University of Chicago Medical Center, Chicago, Illinois; University of Chicago Medical Center, Chicago, Illinois; Advocate Lutheran General, Park Ridge, Illinois; Advocate Lutheran General, Park Ridge, Illinois

## Abstract

**Background:**

*Clostridium difficile* infection (CDI) is the most common cause of nosocomial diarrhea in the United States. This burden is more prevalent and severe in the oncologic population and is associated with greater morbidity and mortality. Diagnostic modalities are limited, and misdiagnosis is high with resultant mismanaged care – inappropriate antibiotics, and potentially missed opportunities for appropriate targeted care towards graft-versus-host disease or other etiologies. To date, no large-scale evaluations have been performed to characterize the incidence of true disease in this population or to help delineate predictive markers for true infections (cell cytotoxicity neutralization assay (CCNA) positivity).

**Methods:**

This is a multi-center, prospective stool banking study from January 2020 to date enrolling pediatric patients with an oncologic diagnosis admitted at Advocate Children’s Hospital and Comer Children’s Hospital. Stool was collected weekly along with relevant clinical histories and symptomology. Isolates of *C. difficile* were cultured and CCNA performed. Stool was also collected during episodes of PCR diagnosed CDI.

**Results:**

65 unique subjects were enrolled over 183 admissions. 11 samples from 10 patients were thought to represent CDI with PCR positivity noted. Five of these specimens were confirmed as CCNA positive while the remainder did not have active toxin presence. Bristol type 4 stools or > 3 stools in 24 hours were significant factors for modeling CCNA positivity, but not for modeling PCR positivity. Notably 26 stool specimens were CCNA positive, and 21 did not have PCR tests done. Among all CCNA positive specimens, no significant difference was noted in symptomatology or antibiotic use.

**Figure:**
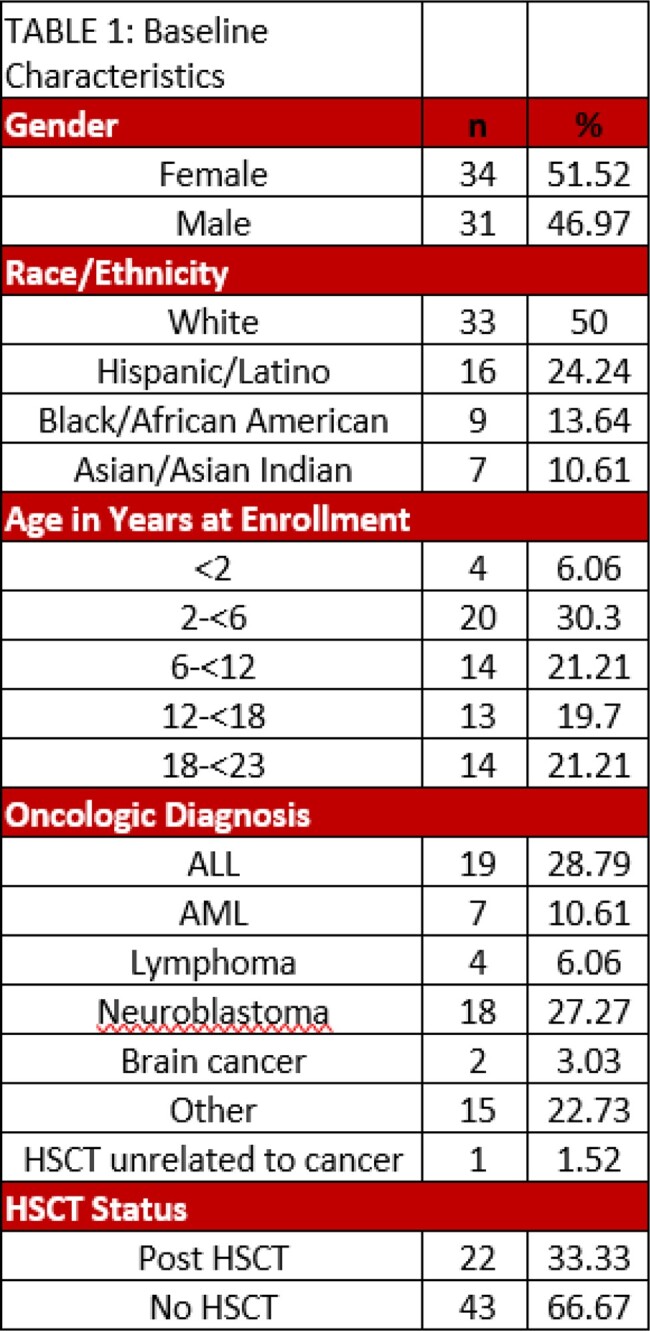

**Figure:**
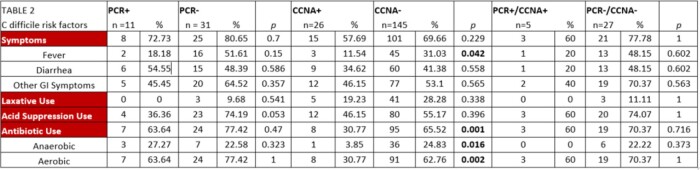

**Figure:**
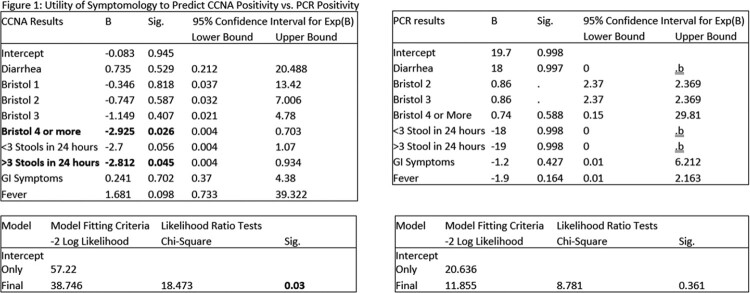

**Conclusion:**

Differentiating true CDI can be challenging. However, even in patients with high risk of alternative GI pathology, the frequency of stooling and stool characteristics can be used. Although considered the gold standard for assessment of disease, CCNA positivity in this population was noted in patients who were not evaluated for disease by their primary providers. Underdiagnosis is possible, but potentially less likely given the absence of clinically significant differentiation in symptoms. Host factors predisposing to disease are likely present.

**Disclosures:**

**Larry K. Kociolek, MD, MSCI**, Merck: Grant/Research Support

